# Efficient Fourth-Order PSTD Algorithm with Moving Window for Long-Distance EMP Propagation

**DOI:** 10.3390/s24196317

**Published:** 2024-09-29

**Authors:** Yongli Wei, Baofeng Cao, Zongxiang Li, Tianchi Zhang, Changjiao Duan, Xiao Li, Xiaoqiang Li, Peng Li

**Affiliations:** 1 State Key Laboratory of NBC Protection for Civilian, Beijing 102205, China; 2 College of Information and Communication Engineering, Harbin Engineering University, Harbin 150001, China

**Keywords:** fourth-order PSTD, moving window, electromagnetic pulse, long-distance propagation, numerical simulation

## Abstract

Satellite-borne electromagnetic pulse (EMP) detection technology plays an important role in military reconnaissance, space environment monitoring, and early warning of natural disasters. However, the complex ionosphere greatly distorts the waveform during propagation and poses a challenge to EMP detection. Therefore, it is necessary to conduct theoretical research on EMP propagation in the ionosphere. Conventional second-order pseudo-spectral time-domain (PSTD-2) algorithm has difficulties in keeping the stability and accuracy of waveforms in calculations over hundreds of kilometers of propagation. To overcome the difficulties, a fourth-order PSTD algorithm incorporating the moving window technique (MWPSTD-4) is proposed. In the numerical examples, the performance of MWPSTD-4 is compared with PSTD-4 and PSTD-2 in the long-distance propagation of EMP. The results show that the MWPSTD-4 improves efficiency while guaranteeing accuracy and is suitable for large-scale electromagnetic field simulation. The proposed method provides a basic algorithm to eliminate the numerical dispersion interference for calculating the long-distance propagation of EMP in complex spaces and is helpful for the design and calibration of satellite-borne EMP detectors.

## 1. Introduction

Satellite-borne detection of Electromagnetic pulse (EMP), such as lightning EMP and nuclear EMP, has been of wide interest [1,2,3,4,5,6,7,8]. As a result of the ionospheric dispersion, the waveforms are severely distorted, which greatly reduces the accuracy of satellite-borne-detected EMP [9]. Consequently, theoretical efforts and experimental work have been conducted to predict and explain waveform distortions of EMP propagation in the ionosphere [10,11,12,13,14,15].

When applied to large-scale spatial problems, the finite-difference time-domain (FDTD) method has the defects of high consumption of computational memory and low efficiency. In 1997, Liu proposed the pseudo-spectral time-domain (PSTD) algorithm. The method employs second-order accurate finite-difference in the time domain, whereas high-precision approximation is achieved by the Fourier transform in the spatial domain, which greatly improves the computational efficiency [16,17,18]. According to the Nyquist sampling theorem, at least two grid points per wavelength provide infinite order accuracy in space, whereas FDTD requires 8–16 grids for the same accuracy, which is 4^D^–8^D^ times more efficient than FDTD (D is the dimension of the problem). Yang Dan et al. [19,20,21,22,23] used the PSTD algorithm to simulate the propagation of EMP in the ionosphere, showing the significant dispersion of the ionosphere on the waveform. However, the numerical dispersion of the PSTD algorithm itself on the calculation results was not fully excluded.

A fourth-order accurate-in-time PSTD algorithm (denoted as PSTD-4) was proposed to further optimize the PSTD algorithm. This method employs the Fourier transform in the spatial domain, while the fourth-order time-accurate difference is applied in the time domain, which greatly reduces the numerical dispersion compared to PSTD-2 [24,25]. Nevertheless, the application of PSTD-4 to long-distance EMP propagation simulations has not yet been validated.

In this paper, we compare the performance of PSTD-4 and PSTD-2 on the propagation over long distances. Moreover, for a propagating pulse, the energy is limited to a localized portion, and the moving window technique combined with PSTD-4, i.e., MWPSTD-4, is introduced to reduce the memory requirement and improve the efficiency. By comparing the performance of PSTD-2, PSTD-4, and MWPSTD-4, the parameters, such as window length and time step, are discussed in terms of dispersion and efficiency. Numerical experiments show that MWPSTD-4 can reduce the calculation time to less than 5 min without dispersion for a 1000 km distance, which can be extended to fast calculations of EMP in the ionosphere over hundreds of kilometers.

This paper is divided as follows: in Section 2, we review the PSTD-4 in detail, giving the stability conditions and the PML absorption boundary format. In addition, the initial-condition excitation technique and the moving window technique are introduced. In Section 3, we systematically evaluate the performance of the PSTD-2, PSTD-4, and MWPSTD-4 algorithms, highlighting the advantages of the MWPSTD-4. In Section 4, we summarize the whole paper and look forward to the potential applications and future directions of the MWP STD-4 algorithm in the field of EMP satellite-based detection.

## 2. Materials and Methods

### 2.1. Maxwell Equations with Fourth-Order Accurate Time-Difference

Taylor-series expansions of a general field component f about time step n yield the following fourth-order accurate finite-difference approximation for the time derivative of f

(1) $$\frac{\partial f}{\partial t}{|}^{n}=\frac{f{|}^{n+1/2}-f{|}^{n-1/2}}{\Delta t}-\frac{\Delta t^{2}}{24}\frac{\partial^{3}f}{\partial t^{3}}{|}^{n}+O\left(\Delta t^{4}\right).$$

Note that if only the first term on the right side is retained, the central difference formula is reduced to second-order accuracy. The Maxwell’s equations are based on the international system of Units. Applying Equation (1) to the time derivatives in Maxwell’s curl equations, we achieve

(2) $$H{|}_{i,j,k}^{n+1/2}=H{|}_{i,j,k}^{n-1/2}-\frac{\Delta t}{\mu_{0}}\left(\nabla \times E{|}_{i,j,k}^{n}\right)+\frac{\Delta t^{3}}{24}\frac{\partial^{3}H{|}_{i,j,k}^{n}}{\partial t^{3}},$$

(3) $$E{|}_{i,j,k}^{n+1}=E{|}_{i,j,k}^{n}+\frac{\Delta t}{\varepsilon_{0}}\left(\nabla \times H{|}_{i,j,k}^{n+1/2}\right)+\frac{\Delta t^{3}}{24}\frac{\partial^{3}E{|}_{i,j,k}^{n+1/2}}{\partial t^{3}}.$$

We convert the time derivatives in Equations (2) and (3) to spatial derivatives using Maxwell’s curl equations as follows:

(4) $${\left.\frac{\partial^{3}H}{\partial t^{3}}\right|}_{i,j,k}^{n}={\varepsilon_{0}c^{4}\left.\nabla \times \nabla \times \nabla \times E\right|}_{i,j,k}^{n},$$

(5) $${\left.\frac{\partial^{3}E}{\partial t^{3}}\right|}_{i,j,k}^{n+1/2}=\mu_{0}c^{4}{\left.\nabla \times \nabla \times \nabla \times H\right|}_{i,j,k}^{n+1/2}.$$

Substituting Equations (4) and (5) into Equations (2) and (3), we obtain a complete PSTD algorithm with fourth-order time stepping:

(6) $${\left.H\right|}_{i,j,k}^{n+1/2}={\left.H\right|}_{i,j,k}^{n-1/2}{-\Delta tc}^{2}\varepsilon_{0}{\left.\nabla \times E\right|}_{i,j,k}^{n}+\frac{\Delta t^{3}c^{4}\varepsilon_{0}}{24}{\left.\nabla \times \nabla \times \nabla \times E\right|}_{i,j,k}^{n},$$

(7) $${\left.E\right|}_{i,j,k}^{n+1}={\left.E\right|}_{i,j,k}^{n}+\Delta tc^{2}\mu_{0}{\left.\nabla \times H\right|}_{i,j,k}^{n+1/2}-\frac{\Delta t^{3}c^{4}\mu_{0}}{24}{\left.\nabla \times \nabla \times \nabla \times H\right|}_{i,j,k}^{n+1/2}.$$

Note that without the last term on the right side, it reduces to the PSTD-2.

### 2.2. Moving Window PSTD-4 (MWPSTD-4)

#### 2.2.1. PSTD with Fourth-Order Accurate Time Stepping

For the PSTD algorithm, we use the discrete Fourier transforms to evaluate the spatial derivatives on an unstaggered, collocated grid. The z derivative of a general field component f that is known at all grid points i along the z direction can be computed as follows:

(8) $${\left.\frac{\partial f}{\partial z}\right|}_{i}\approx {F_{z}}^{-1}[-jk_{z}F_{z}\left(\begin{array}{c} f) \end{array}\right]{|}_{i},$$

where $k_{z}$ is the Fourier-transform variable representing the z component of the numerical wave vector, and $F_{z}$ and ${F_{z}}^{-1}$ denote, respectively, the forward and the inverse discrete Fourier transforms along the z direction. According to the Nyquist sampling theorem, a sampling density of at least two grids per wavelength can be achieved with infinite precision in space. Hence, phase velocity errors in the PSTD algorithm arise only from the second-order time difference. In the 1-D case with $E_{x}$ polarization and z-directed propagation, Equations (6) and (7) can be written as

(9) $$H_{y}{|}_{k}^{n+1/2}=H_{y}{|}_{k}^{n-1/2}-\Delta tc^{2}\varepsilon_{0}\left\{F_{z}^{-1}\left[-jk_{z}F_{z}\left(E_{x}{|}_{k}^{n}\right)\right]\right\}-\frac{\Delta t^{3}c^{4}\varepsilon_{0}}{24}\left\{F_{z}^{-1}\left[-jk_{z}^{3}F_{z}\left(E_{x}{|}_{k}^{n}\right)\right]\right\},$$

(10) $${\left.E_{x}\right|}_{k}^{n+1}={\left.E_{x}\right|}_{k}^{n}-\Delta tc^{2}\mu_{0}\left\{F_{z}^{-1}\left[-jk_{z}F_{z}\left(H_{y}{|}_{k}^{n+1/2}\right)\right]\right\}-\frac{\Delta t^{3}\mu_{0}c^{4}}{24}\left\{F_{z}^{-1}\left[-jk_{z}^{3}F_{z}\left(H_{y}{|}_{k}^{n+1/2}\right)\right]\right\}.$$

Note that without the last term on the right side in Equations (6) and (7), it reduces to the PSTD-2. The stability criterion of PSTD-2 and PSTD-4 both satisfies

(11) $$\Delta t\leq \frac{2\Delta z}{c\sqrt{D}\pi }.$$

#### 2.2.2. Perfectly Matched Layer

The wraparound effect, a major limitation caused by the periodicity assumed in the FFT, is removed using Berenger’s perfectly matched layers. In the 1-D case, the updating equations in the PML region are given by

(12) $${\left.H_{y}\right|}_{k}^{n+1/2}=\frac{2\mu_{0}-\sigma_{m}\Delta t}{2\mu_{0}+\sigma_{m}\Delta t}{\left.H_{y}\right|}_{k}^{n-1/2}-\frac{2\Delta t}{2\mu_{0}+\sigma_{m}\Delta t}F_{z}^{-1}\left[-jk_{z}F_{z}\left(E_{x}{|}_{k}^{n}\right)\right],$$

(13) $${\left.E_{x}\right|}_{k}^{n+1}=\frac{2\varepsilon_{0}-\sigma \Delta t}{2\varepsilon_{0}+\sigma \Delta t}{\left.E_{x}\right|}_{k}^{n}-\frac{2\Delta t}{2\varepsilon_{0}+\sigma \Delta t}F_{z}^{-1}\left[-jk_{z}F_{z}\left(H_{y}{|}_{k}^{n}\right)\right].$$

where σ represents the conductivity within the PML region and satisfies

(14) $$\sigma \left(\rho \right)=\sigma_{max}{\left(\rho /d\right)}^{n}.$$

ρ is the distance between the current position and the outer boundary of the PML; d is the thickness of the PML region; and n is the PML type coefficient. When n=1, σ varies linearly; when n=2, σ varies parabolically. The conductivity and magnetic permeability satisfy

(15) $$\frac{\sigma }{\varepsilon_{0}}=\frac{\sigma_{m}}{\mu_{0}}.$$

#### 2.2.3. Initial-Condition Excitation Technique

In the PSTD algorithm, if a hard excitation source is introduced, an unwanted Gibbs phenomenon will appear when applying the fast Fourier transform (FFT) to represent spatial derivatives. The initial-condition excitation technique is used to simulate the incidence of a 1-dimensional plane wave [26]. The electric field $E_{x}^{0}$ and the magnetic field $H_{y}^{0.5}$ of Gaussian pulse initially distributed in the computational domain are expressed as

(16) $$E_{x}^{0}\left(i\right)=E_{0}\exp\left(\xi \frac{(i-i_{c}{)}^{2}}{i_{p}^{2}}\right),$$

(17) $$H_{y}^{0.5}\left(i\right)=\frac{E_{0}}{Z_{0}}\exp\left(\xi \frac{(i-i_{c}-0.5\Delta z/c\Delta t{)}^{2}}{i_{p}^{2}}\right).$$

where $E_{0}$ is the amplitude of the Gaussian pulse initially peaked at grid $i_{c}$; $i_{p}$ denotes the pulse width defined at the positions where the peak value falls to the value of 0.001$E_{0}\left(\xi =ln(0.001)\right)$; $Z_{0}$ is the impedance of the free space, and c is the speed of wave propagation. Note that we have introduced a time-delay term, 0.5Δz/cΔt, in Equation (17), considering that the magnetic field lags behind the electric field by a half-time step.

#### 2.2.4. Moving Window Technique

**Figure 1:**
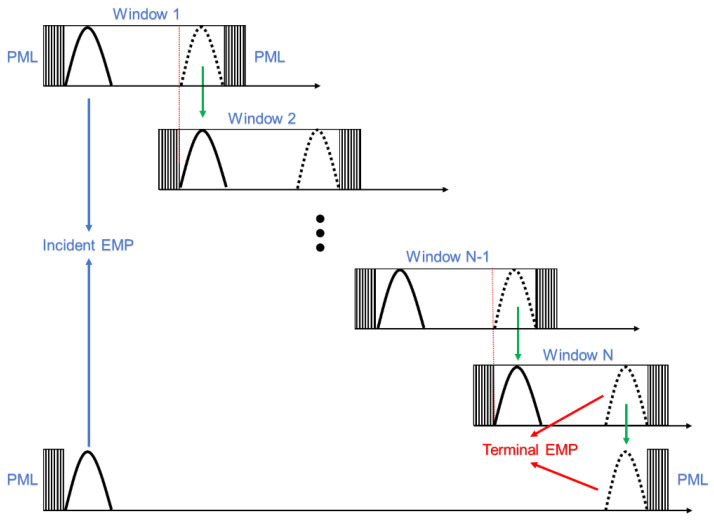
Illustration of moving window concept.

A straightforward application of PSTD to propagation is very wasteful of computer memory since, for a propagating pulse, the energy is essentially confined to a localized portion of the entire path. All the PSTD mesh that is outside of the region containing the significant pulse energy is wasted memory and calculation effort. Since the FFT in the PSTD algorithm requires all spatial points, the memory and computation time required increases as the length of the problem space increases. In addition, the spatial locations at which the waveform has not arrived are meaninglessly involved in the global transformation of the FFT. Therefore, PSTD-4 incorporating the moving window technique [27,28,29] (denoted by MWPSTD-4) is proposed to perform EMP propagation calculations. Both sides of the moving window are set as PML region. When the EMP propagates to the right side of the window, the pulse is recorded, and the window is then moved forward a distance so that the PML region at the left end of the window meets the tail of the wave, and the calculation continues. This operation is repeated until the pulse propagates to the given distance, as shown in Figure 1.

## 3. Results and Discussion

### 3.1. Comparison of the Numerical Accuracy of PSTD-2 and PSTD-4

In this section, we analyze the numerical accuracy of PSTD-2 and PSTD-4 algorithms. On the one hand, the reason why the numerical dispersion appears is that the partial derivative is replaced by difference. The more items are retained, the more accurate the calculation result will be. According to Equation (1), fourth-order accurate finite-difference approximation for the time derivative perserves one more term $-\frac{\Delta t^{2}}{24}\frac{\partial^{3}f}{\partial t^{3}}{|}^{n}$ than second-order accuracy, which reduces the truncation error of MWPSTD-4.

On the other hand, the dispersion relations of PSTD-2 and PSTD-4 are different. The numerical dispersion relation for PSTD-2 is

(18) $$k=\frac{2}{c\Delta t}\sin\left(\frac{\omega \Delta t}{2}\right).$$

where k represents the numerical wave number, while for PSTD-4, the dispersion relation is

(19) $$k[1-(\frac{c^{2}\Delta t^{2}}{24})k^{2}]=\frac{2}{c\Delta t}sin(\frac{\omega \Delta t}{2}).$$

**Figure 2:**
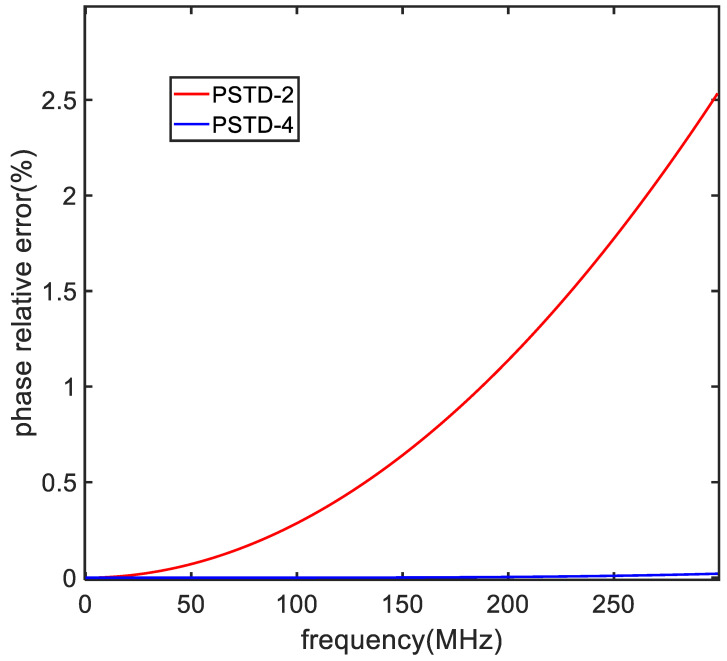
Comparison of the free-space numerical phase velocity errors associated with the PSTD-2 and PSTD-4 methods.

To compare the numerical dispersion properties of PSTD-2 and PSTD-4, we compute the numerical phase velocity using Equations (18) and (19). The frequency ranges from dc to 300 MHz for both methods. The Δz is set as 0.25 m, which is a quarter of the max wavelength. The time step satisfies the stability criterion and is set as 4.16 × 10^−10^ s. The error in the numerical phase velocity is plotted in Figure 2. At this time step, the numerical phase velocity errors are 2.5% and 0.02% at 300 MHz associated with PSTD-4 and PSTD-2, respectively.

### 3.2. Comparison of Stability and Efficiency of PSTD-2 and PSTD-4

**Figure 3:**
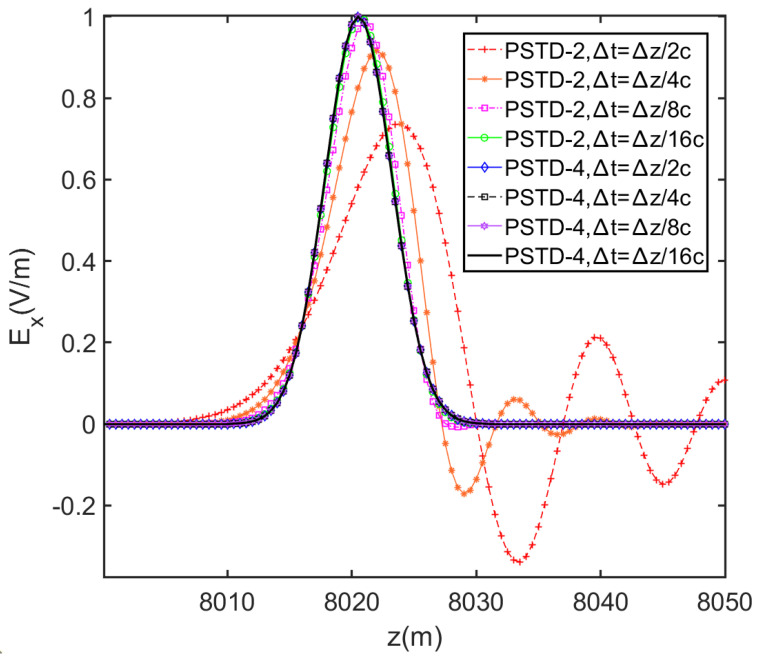
Gaussian waveforms after propagating 8 km.

In this section, we present the results of numerical experiments designed to test the performance of the PSTD-2 and PSTD-4 algorithms in long-distance propagation computations. The distance is set to 8 km. A Gaussian pulse with a maximum frequency of 300 MHz is chosen as the source excitation, with a maximum peak value of 1 V/m. The spatial grid cell size is chosen to be $∆z=\lambda_{min}/2=0.5\;m$, and the time steps are set as ∆t=∆z/2c, ∆t=∆z/4c, ∆t=∆z/8c, and ∆t=∆z/16c for both PSTD-2 and PSTD-4. Figure 3 and Table 1 show the waveforms after propagation and the CPU time spent, respectively. The computer configuration is as follows: CPU is 13th Gen Intel Core i9-13900K, and the Random Access Memory is 64 GB.

First, we compare the numerical dispersion of PSTD-2 and PSTD-4. No numerical dispersion exists in the PSTD-4 algorithm when the time steps are set to the above time steps. However, the PSTD-2 algorithm can suppress the numerical dispersion only when the time step is reduced to ∆t=∆z/16c, and the distortion increases significantly with the increase in the time step. This indicates that PSTD-4 has higher computational efficiency in EMP propagation. Second, we investigate the correlation between computational efficiency and time steps. The CPU time of the PSTD-2 and PSTD-4 at different time steps (∆t=∆z/2c to ∆t=∆z/16c) shows an almost two-fold trend increment. Although CPU time (27 s) of PSTD-4 with ∆t=∆z/2c is twice as much as that of PSTD-2 at the same time step, this time, cost is exchanged for an accurate solution with no numerical dispersion, which reflects the positive impact of algorithm optimization. Third, without numerical dispersion, the PSTD-2 algorithm needs to set the time step to ∆t=∆z/16c, and the time spent is 120 s. In contrast, the PSTD-4 algorithm only needs 27 s at ∆t=∆z/2c, saving 77.5% of the time. These results demonstrate that compared to PSTD-2, PSTD-4 is dispersion-free and time-saving in long propagation calculation.

### 3.3. Comparison of Efficiency and Moving Window Length of PSTD-4 and MWPSTD-4

**Figure 4:**
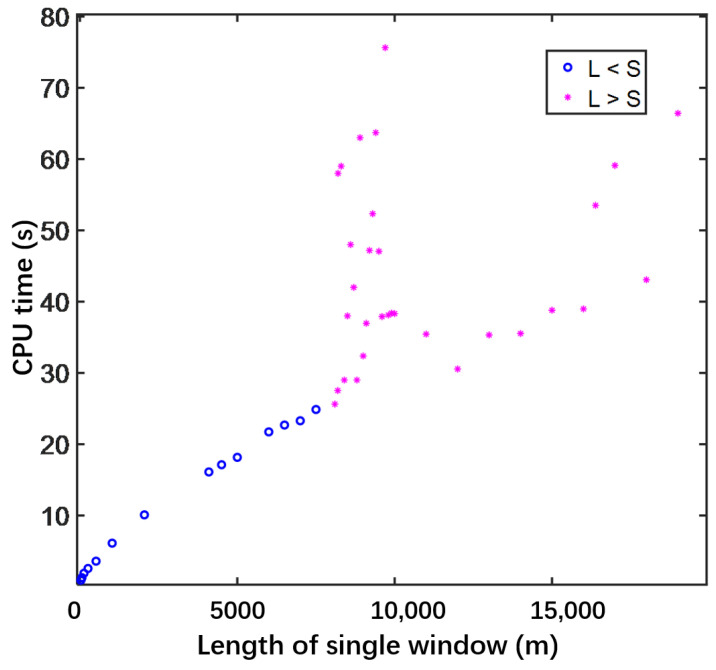
Correlation between CPU time and length of moving window.

To further improve the computational efficiency, we proposed the MWPSTD-4 algorithm in this paper. In this section, we present the results of numerical experiments designed to test the performance of the PSTD-4 and MWPSTD-4 algorithms in long-distance computations. We study the correlation between the moving window length and CPU time. The Gaussian waveform parameters adopted in this section are consistent with those in Section 3.2; the results are shown in Figure 4.

On the one hand, if the window length L is smaller than the propagation distance S, the simulation requires at least two windows, which is denoted by “○” in Figure 4. The smaller the window length, the more windows are required, but less CPU time is needed for propagation. On the other hand, if window length L is larger than propagation distance S, only one window is needed, denoted by “✳” in Figure 4. The CPU time for propagation has an overall increasing trend as the window length increases. However, there is also a lower limit on the size of the moving window, which must be able to accommodate a complete Gaussian pulse waveform.

### 3.4. MWPSTD-4 Applied in Long-Distance Propagation

**Figure 5:**
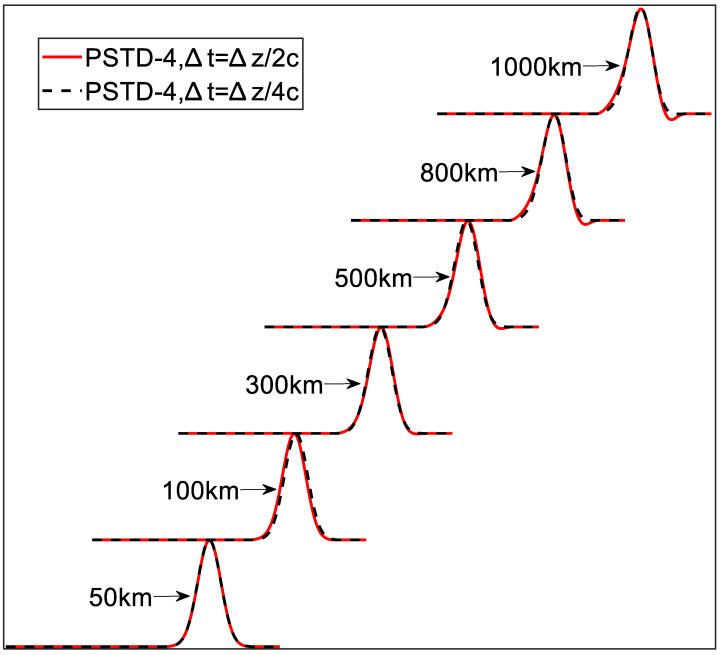
Waveforms after propagating the same distance at different time steps.

In this section, the MWPSTD-4 is applied to calculate the Gaussian pulse propagating to different distances. The waveform parameters adopted in this section are consistent with those of PSTD-4 in Section 3.2. Yet, the time steps are set as ∆t=∆z/4c and ∆t=∆z/2c. Figure 5 and Table 2 show the waveforms after propagation and the CPU time required, respectively.

The results show that ∆t=∆z/4c takes twice as long as ∆t=∆z/2c for the same distance. When the time step is set as ∆t=∆z/4c, the waveform has no dispersion for propagation up to even 1000 km. However, when the time step is set as ∆t=∆z/2c, the waveforms do not show dispersion until 500 km, and the dispersion is more obvious at 1000 km. Actually, such a slight dispersion on the 1000 km propagation calculation has little influence on results compared to the real-time domain waveform detected [1,2].

This phenomenon can be explained by the fact that the fourth-order accurate difference is still essentially a truncation of the Taylor equation and, therefore, inevitably introduces a truncation error, which accumulates and becomes visible when propagated to a certain distance. By appropriately reducing the time step, the cumulative effect of this error can be suppressed to a certain extent so as to maintain high waveform accuracy over longer propagation distances. These validate the efficiency of the MWPSTD-4 method in dealing with long-range propagation problems.

## 4. Conclusions

In this paper, we propose a fourth-order pseudo-spectral time-domain algorithm incorporating the moving window technique to address the numerical dispersion issue encountered in the PSTD-2 algorithm during the simulation of long-distance EMP propagation.

Initially, we derive the general formulations of both PSTD-2 and PSTD-4 algorithms and evaluate their performance by comparing the waveforms and the CPU time following the propagation of a Gaussian pulse. We prove the superiority of PSTD-4 in accuracy from the expression of the algorithm and the dispersion relation. The results indicate that the PSTD-4 algorithm effectively mitigates numerical dispersion during long-distance propagation while improving efficiency.

Then, we propose the moving window PSTD-4 (MWPSTD-4) algorithm to simulate EMP propagation over varying distances and delve into the correlation between the moving window length and the corresponding CPU time by systematically adjusting the window size. Our analysis reveals that shortening the window length can significantly reduce the computational cycle, ensuring stability and accuracy. The results demonstrate that by fine-tuning the time-step size, we can effectively curb the cumulative effect of errors, preserving high waveform accuracy over extended propagation distances.

The proposed algorithm not only eliminates potential distortions introduced by numerical dispersion but also offers a viable solution for simulating EMP propagation in intricate spatial environments.

## Figures and Tables

**Table 1:** CPU time and time step when Gaussian waveform propagates 8 km.

Method Type	Time Step	CPU Time
PSTD-4	∆t=∆z/2c	27 s
PSTD-4	∆t=∆z/4c	52 s
PSTD-4	∆t=∆z/8c	101 s
PSTD-4	∆t=∆z/16c	202 s
PSTD-2	∆t=∆z/2c	15 s
PSTD-2	∆t=∆z/4c	30 s
PSTD-2	∆t=∆z/8c	60 s
PSTD-2	∆t=∆z/16c	120 s

**Table 2:** Correlation between CPU time and propagation distance at different time steps.

Distance	Time Step	CPU Time
50 km	∆t=∆z/2c	7.54 s
	∆t=∆z/4c	15.14 s
100 km	∆t=∆z/2c	15.16 s
	∆t=∆z/4c	30.12 s
300 km	∆t=∆z/2c	45.45 s
	∆t=∆z/4c	89.94 s
500 km	∆t=∆z/2c	75.22 s
	∆t=∆z/4c	150.07 s
800 km	∆t=∆z/2c	120.32 s
	∆t=∆z/4c	240.16 s
1000 km	∆t=∆z/2c	150.48 s
	∆t=∆z/4c	302.79 s

## Data Availability

Data are contained within this article.
